# A comprehensive comparison between Terahertz and optical wireless communications

**DOI:** 10.1038/s44459-025-00002-1

**Published:** 2025-11-06

**Authors:** Mingqing Liu, Hossein Kazemi, Majid Safari, Iman Tavakkolnia, Harald Haas

**Affiliations:** 1 College of Electronics and Information Engineering, Tongji University, Shanghai, China; 2 LiFi Research and Development Centre, Electrical Engineering Division, University of Cambridge, Cambridge, UK; 3 Institute for Imaging, Data, and Communication, School of Engineering, University of Edinburgh, Edinburgh, UK

**Keywords:** Mathematics and computing, Information technology

## Abstract

This paper presents a comprehensive quantitative comparison between Terahertz (THz) communication and optical wireless communication (OWC) technologies, with a focus on indoor and outdoor deployment scenarios. For indoor environments, we compare THz and vertical-cavity surface-emitting laser (VCSEL)-based OWC systems by incorporating misalignment effects using a multi-ray THz channel model with antenna patterns and a Gaussian beam model for VCSELs. Unified beamwidth assumptions enable consistent evaluation. We further develop power consumption models capturing THz phase noise, VCSEL nonlinearities, and photodetector bandwidth-area tradeoffs, facilitating a detailed energy efficiency analysis under multi-transmitter coverage. For outdoor scenarios, we survey existing stochastic channel models that characterize path loss, pointing errors, and small-scale fading for both THz and free space optics (FSO) links. These models are applied to unmanned aerial vehicle (UAV)-based use cases to evaluate communication robustness under dynamic conditions. Our findings highlight critical performance tradeoffs and deployment challenges, offering insights into the relative advantages of THz and OWC technologies in diverse environments.

## Introduction

The rapid growth in demand for high-speed, high-capacity wireless communication has driven interest in Terahertz (THz) and optical wireless communication (OWC) technologies, each offering unique strengths for short- and long-distance links^1,2^. THz links, operating in the high-frequency spectrum from 0.1 THz to 1 THz, and OWC, leveraging visible and infrared light, offer promising alternatives to traditional RF technologies, especially in scenarios where bandwidth or interference constraints are critical^3^. From existing experimental demonstrations, both technologies are capable of supporting multi-kilometer Tbit/s wireless data transmission^4,5^. Still, comparing these technologies is essential to identify their respective strengths and limitations in addressing diverse communication needs. On the one hand, the comparison of transmission characteristics of THz and optical wireless technologies enables us to find appropriate application scenarios for each technology. On the other hand, the performance gap and corresponding impact factors of THz and optical wireless in a common application scenario still need demonstration to date. Thus, this manuscript aims to provide a comprehensive comparison of these two technologies.

Most existing comparative studies between THz and OWC technologies are primarily qualitative, providing general insights into their characteristics but lacking quantitative performance assessments^6–8^. Specifically, ref. ^9^ and ref. ^10^ present comprehensive comparisons of wireless communication across different spectrum bands, including sub-6 GHz, mmWave, THz, and optical frequencies. These works highlight architectural considerations, resource allocation, and tradeoffs involved in multi-band networking. Furthermore, ref. ^11^ focuses on hybrid THz / visible light communication (VLC) network design and mobility-aware resource allocation in indoor scenarios. While these studies offer valuable system-level perspectives, they do not provide a detailed, channel-specific quantitative comparison between THz and OWC technologies, particularly under dynamic conditions such as pointing errors and environmental impairments.

However, quantitative analysis is crucial for understanding the practical viability of these technologies under specific conditions, such as power efficiency, alignment precision, and environmental impacts. Quantitative comparisons can provide more concrete guidance for technology selection and optimization in real-world deployments. OWC can be categorized into short-range indoor OWC, often referred to as LiFi, and long-distance free space optics (FSO)^12^. Similarly, THz links are divided into indoor and outdoor applications to address distinct propagation characteristics in each scenario^3,13^. In our previous work, we conducted a comparative study of the two technologies, focusing on point-to-point links in indoor environments^14^. Thus, this paper conducts quantitative analyses for both indoor and outdoor environments, further incorporating an accurate THz multi-path channel model and a network-level system evaluation indoors, thereby enabling a more comprehensive comparison of THz and OWC performance under varying conditions.

In indoor settings, vertical-cavity surface-emitting laser (VCSEL)-based OWC is regarded as a potential “LiFi 2.0” solution^15^, while electronics-based THz technology offers a cost-effective and mature alternative compared to photonic- and plasmonic-based THz technologies^16^. Thus, we focus our indoor comparison on these two systems. To mitigate the significant path loss at high frequencies, both THz and OWC systems require directional transmission with energy-concentrating beams, making misalignment analysis essential. Additionally, energy efficiency is a critical factor in indoor scenarios, leading us to establish channel models that account for misalignment effects and develop energy consumption models for a detailed numerical analysis^17–19^. Through quantitative analysis, we demonstrate that VCSEL-based OWC outperforms Terahertz communication (TeraCom) in energy efficiency but is more sensitive to transmission distance and bandwidth limitations. Consequently, VCSEL-based OWC requires a more focused beam to mitigate these challenges effectively.

For outdoor environments, extensive research has been conducted on stochastic channel models for FSO^20,21^ and THz links^22,23^. These models describe essential characteristics, including THz absorption properties and the sensitivity of FSO to turbulence and weather conditions, as well as the pointing error characteristics of both channels. Summarizing and analyzing these models through numerical comparisons can reveal key similarities and differences between FSO and THz links. Furthermore, compared to terrestrial applications where transceivers are typically fixed or slowly varying, unmanned aerial vehicle (UAV)-based communication introduces unique challenges, particularly in terms of pointing errors due to continuous mobility and vibrations. Our study focuses on UAV scenarios to especially highlight the impact of misalignment on system performance, as this factor is far more pronounced and dynamic than in ground-based settings. Applying these models to UAV scenarios, which are highly sensitive to pointing errors, can provide valuable insights into the suitability of each technology for mobile outdoor applications, helping identify the optimal solution for dynamic and challenging environments^24–26^. Numerical results from the comparative analysis reveal the impact of weather conditions, absorption, and pointing errors on both links. Besides, FSO is more sensitive to transmission distance and misalignment, making it less suitable for UAV-to-UAV applications compared to THz links. The contribution of this manuscript is summarized as follows.

1. We propose a method to compare TeraCom and VCSEL-based OWC in an indoor environment with the misalignment effect. For TeraCom, we incorporate the antenna radiation pattern with pointing directions into a multi-ray THz channel model, capturing the effects of directional beam propagation. For VCSEL-based OWC, we employ a Gaussian beam model that accounts for geometric misalignment. By unifying the beamwidth parameters of the THz and optical antennas, this approach enables a quantitative analysis of each system’s performance and the impact of misalignment on signal quality.
2. We develop power consumption models for TeraCom and VCSEL-based OWC, incorporating key factors such as cascaded component power efficiency and phase noise for THz systems, and non-linear conversion effects of VCSELs, along with the bandwidth-area tradeoff of photodetectors. Using these analytical models alongside channel models, we perform a numerical analysis to compare the energy efficiency of both systems. Additionally, we extend this analysis to an indoor environment with multiple transmitters to evaluate coverage and service quality.
3. We summarize existing stochastic channel models for FSO and THz links, covering path loss, pointing error, and small-scale fading, to assess link performance in outdoor environments with various environmental conditions. In particular, we compare two different pointing error models for THz channels. Finally, we apply these channel models to UAV applications to evaluate link performance in dynamic scenarios.

The remainder of this manuscript is organized as follows. The “Results” section presents comparative results for THz and OWC technologies in both indoor and outdoor environments. The “Discussion” section provides a brief summary of key findings and outlines potential directions for future work. The “Methods” section details the analytical models and evaluation methods used, including misalignment modeling, energy efficiency analysis, and stochastic channel characterization for UAV scenarios. Abbreviations used in this paper are listed in Table 1.

**Table 1:** List of abbreviations used in this paper.

Abbreviation	Full form
ADC	Analog-to-Digital Converter
AoA	Angle of Arrival
APs	Access Points
AWGN	Additive White Gaussian Noise
BPF	Band-Pass Filter
CMOS	Complementary Metal–Oxide–Semiconductor
CPC	Compound Parabolic Concentrator
DAC	Digital-to-Analog Converter
DC	Direct Current
DCO-OFDM	DC-biased Optical Orthogonal Frequency Division Multiplexing
DoF	Degrees of Freedom
EM	Electromagnetic
FOV	Field of View
FSO	Free-Space Optics
G2U	Ground-to-UAV
GSCMs	Geometry-based Stochastic Channel Models
HPBW	Half-Power Beamwidth
HPBD	Half-Power Beam Divergence
IF	Intermediate Frequency
IM/DD	Intensity-Modulation/Direct-Detection
LD	Laser Diode
LED	Light-Emitting Diode
LIV	Light–Current–Voltage
LO	Local Oscillator
LoS	Line-of-Sight
MAL	Molecular Absorption Loss
MIMO	Multiple-Input Multiple-Output
NLOS	Non-Line-of-Sight
OWC	Optical Wireless Communication
PA	Power Amplifier
PD	Photodiode
PDF	Probability Density Function
PN	Phase Noise
PV	Photovoltaic
RT	Ray Tracing
SD	Standard Deviation
SINR	Signal-to-Interference-plus-Noise Ratio
SISO	Single-Input Single-Output
SL	Spreading Loss
SLIPT	Simultaneous Lightwave Information and Power Transfer
SNR	Signal-to-Noise Ratio
THz	Terahertz
TIA	Trans-Impedance Amplifier
TeraCom	Terahertz communication
U2G	UAV-to-Ground
U2U	UAV-to-UAV
UAV	Unmanned Aerial Vehicle
ULA	Uniform Linear Array
VCSEL	Vertical-Cavity Surface-Emitting Laser

## Results

### Comparative overview of THz and optical wireless systems

**Fig. 1:**
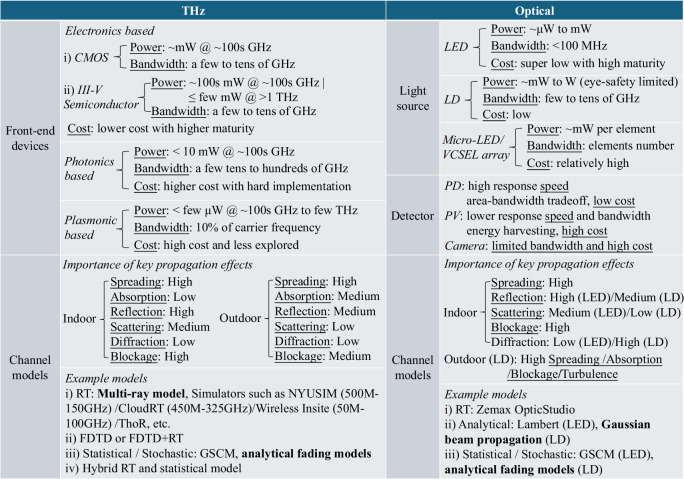
Comparative overview of THz and optical wireless communications (bold terms are the channel modelling methods adopted in this work).

This section presents a comparative overview of THz and optical wireless communication systems, focusing on two fundamental components: transceiver architecture and channel models. This comparison is motivated and informed by prior review studies on the respective technologies^3,12,27–29^. In wireless communication systems, the transceiver architecture forms the foundation, where THz systems typically incorporate front-end electronic devices, while optical systems rely on light sources and detectors. In addition, channel modeling plays a crucial role in evaluating system performance. Therefore, we compare these two technologies from the perspectives of transceiver design and channel characteristics, with particular emphasis on transmitted power, bandwidth, cost, and propagation behavior in both indoor and outdoor environments, as in Fig. 1.

The transceiver architecture of THz systems can be categorized into electronics-based, photonics-based, and plasmonic-based front-end devices. Electronics-based designs, including complementary metal-oxide-semiconductor (CMOS) and III–V semiconductor technologies, offer relatively mature and cost-effective solutions with transmitted power ranging from milliwatts to hundreds of milliwatts and bandwidths up to several tens of GHz^30,31^. Photonics-based THz systems achieve broader bandwidths (up to hundreds of GHz) but face challenges in lower transmitted power, high cost, and implementation complexity^32^. Plasmonic-based designs remain largely unexplored due to high cost and limited maturity, though they offer theoretical potential at frequencies up to several THz^33^. Besides, THz employs horn antennas with gains up to 55 dBi, but their bulkiness limits mobile applications. THz antenna arrays are still under development, particularly at higher frequencies, where precise beamforming remains challenging^34^.

While the hardware for TeraCom systems still requires further effort, OWC technologies primarily employ publicly accessible light sources, such as light-emitting diodes (LEDs) and laser diodes (LDs) in the transmitter, and photodiodes (PDs) and photovoltaic (PV) cells in the receiver^2^. LEDs are widely available and low-cost but are limited in bandwidth (<100 MHz) and output light intensity, which can restrict data rates and coverage. LDs, especially VCSELs, provide higher efficiency and bandwidth (tens of GHz), though they are constrained by eye-safety limits^35,36^. Micro-LED and VCSEL arrays deliver moderate power and high bandwidth, depending on element numbers, with relatively high cost, and are often suitable for multi-element deployment. On the receiver side, PDs and PV cells offer high response speeds and can be adapted for simultaneous lightwave information and power transfer (SLIPT)^12^. However, there is a tradeoff between bandwidth and detection area, affecting the field of view (FOV) and signal sensitivity. Camera-based detectors, while offering larger FOVs, tend to operate at lower speeds and thus are limited to lower-data-rate applications.

We compare the key propagation effects of THz and optical technologies across different application scenarios, highlighting the distinct factors influencing each system. In indoor environments, THz systems are particularly sensitive to spreading loss, reflection, and blockage, due to their relatively short wavelengths and limited diffraction^28^. In outdoor scenarios, in addition to spreading loss, THz propagation is strongly affected by atmospheric absorption, which depends on air composition and humidity, especially near molecular absorption lines^37^. In contrast, indoor OWC behavior varies with the light source. LED-based systems exhibit broader beam divergence and stronger non-line-of-sight (NLOS) reflections^38^, whereas LD-based systems generate narrower, more directed beams, resulting in minimal multi-path reflections^36^. In outdoor OWC, turbulence becomes a dominant propagation impairment due to the short optical wavelengths, which are highly susceptible to small-scale variations in refractive index, leading to scintillation and beam distortion. THz signals, with wavelengths ranging from hundreds of micrometers to several millimeters, are far less affected by such fluctuations and typically neglect turbulence as demonstrated by experiments^39^.

Channel modeling remains a fundamental challenge for both THz and optical wireless systems due to the complex propagation characteristics in these bands. In THz communications, existing surveys typically classify channel models into three categories^3,28,40^: deterministic, statistical, and hybrid models. Ray-tracing (RT) and full-wave methods such as finite-difference time-domain (FDTD) or combined FDTD+RT are widely used among deterministic models. RT-based approaches, including the widely adopted multi-ray model proposed by Chong et al.^41^, offer high accuracy and are especially suitable for detailed indoor scenarios. Several simulators, such as NYUSIM, Wireless Insite, CloudRT, and ThoR^28^ have been developed to support these models. Statistical models, on the other hand, aim to capture large- and small-scale fading characteristics with lower computational complexity. Geometry-based stochastic channel models (GSCMs) are often employed, particularly in indoor-to-outdoor extensions. In addition, numerous studies have modeled small-scale fading as probability density functions (PDFs), enabling analytical derivations of channel capacity and outage performance, which are especially suitable for outdoor scenarios due to the statistical nature of the environment^22,23^. For optical wireless systems, RT and GSCM are widely used for LED-based indoor channels^42,43^. For laser-based systems, which generate narrower beams, Gaussian beam propagation models are more appropriate. Notably, Hossein et al.^36^ developed a misalignment model based on Gaussian beam optics that incorporates receiver displacements and beam divergence. In outdoor FSO communication, analytical fading models based on PDFs are also prevalent, capturing effects such as atmospheric turbulence, pointing errors, and aperture averaging^20,21^. Our earlier work used a stochastic channel model for the THz system indoors to maintain generality^14^. However, to better align with the accurate power and misalignment effect comparisons in this work, we adopt a multi-ray model for THz indoor channels. Moreover, this work further extends the comparison by including outdoor channel models for both technologies, where key propagation impairments, such as absorption for THz and turbulence for OWC, diverge significantly.

Mobility solutions in THz and OWC are at different maturity levels. THz systems are exploring beamforming and multiple-input-multiple-output (MIMO) techniques, but practical deployment remains challenging due to hardware and alignment complexities^44^. In OWC, optical beam steering and MIMO techniques enable mobility^36^, but beam steering is costly, and expanding receiver FOV requires further development^45^. Besides, both systems share challenges in digital backend processing^3^. As required data rates increase, the size, cost, and thermal requirements of digital-to-analog converters (DACs) and analog-to-digital converters (ADCs) become bottlenecks, necessitating efficient, low-power signal processing.

### Numerical comparison in an indoor environment

For performance comparison of TeraCom and VCSEL-based OWC systems in indoor applications, a room with dimensions of 3 m × 3 m × 3 m is configured. The transmitter (Tx) is mounted on the ceiling at a height of 2.95 m, and the receiver (Rx) is assumed to be placed at a height of 0.95 m, as the user equipment is generally on top of tables, etc. Hence, without specifying, the location of Tx is [1.5, 1.5, 2.95] and Rx is [1.5, 1.5, 0.95], respectively, so the link length of both systems is 2 m. Also, the Tx is facing downward and Rx vice versa. For single-input-single-output (SISO) links, the horn antenna is employed in the system, and the half-power beamwidth (HPBW) of the radiated pattern in the azimuth and elevation is assumed to be identical, i.e., ${\theta }_{{\rm{a}}}^{{\rm{bw}}}={\theta }_{{\rm{e}}}^{{\rm{bw}}}$ at both Tx and Rx sides (see Eq. (16) in the “Methods” section). Besides, considering the orientation of Tx and Rx, ${\theta }_{{\rm{a}}}^{0}={0}^{\circ }$ and ${\theta }_{{\rm{e}}}^{0}=9{0}^{\circ }$ are set on the Tx side, while ${\theta }_{{\rm{a}}}^{0}={0}^{\circ }$ and ${\theta }_{{\rm{e}}}^{0}=-9{0}^{\circ }$ are set at the Rx side. For VCSEL-based OWC, a wavelength of 940 nm is selected due to the high manufacturing maturity and widespread commercial availability of VCSELs at this wavelength^46^, while for TeraCom, 350 GHz is chosen to align with established channel modeling studies and ensure modeling accuracy^41^.

To obtain the channel gain of the TeraCom system, a multi-ray channel simulation follows the logic in ref. ^41^, where the key parameters for calculating *R*(*f*), *S*(*f*), *L*(*f*), which denote reflection, scattering, and diffraction coefficients for a rough surface, can be found. Additionally, we include the double-bounce reflection and scattering effect, neglecting the diffraction effect in indoor scenarios at the 350 GHz band. The parameters for evaluating TeraCom’s signal-to-noise ratio (SNR) and consumption factor (CF), i.e., noises, gain/efficiency of key components, in the transceiver architecture are listed in Table 2. To numerically analyze the performance of VCSEL-based OWC, receiver-related parameters are from ref. ^36^, and the parameters for the VCSEL conversion process are obtained through the fitting process of measurement results^46^. The parameters for the two systems used in the numerical analysis are summarized in Table 2, and the commonly adopted parameters include: temperature in Kelvin, *K* = 295, power consumed by the signal processing components *P*_others_ = 100 mW. In the following, we will first compare the two systems by SNR and CF for SISO links in a room. Then, we will analyze coverage probability as well as CF of the two systems when multiple Txs are deployed. All simulations are conducted in MATLAB.

**Table 2:** Parameters for evaluation in indoor scenarios

TeraCom			VCSEL-based OWC		
Parameter	Symbol	Value	Parameter	Symbol	Value
Frequency	*f*	350 GHz	Wavelength	*λ*	940 nm
-	-	-	Original beam waist	*ω*_0_	2.523 μm
PN floor	*K*_0_	−110 dBc/Hz	Relative intensity noise	*N*_RI_	−155 dB/Hz
Rx noise figure	*F*	10.6 dB	TIA noise figure	*F*_n_	5 dB
Gain of PA	*G*_PA_	10.9 dB	PD responsivity	*R*_PD_	0.6 A/W
Power efficiency of PA	*η*_PA_	0.1165	Load resistance	*R*_L_	50 *Ω*
Gain/efficiency of mixer	*G*_M_/*η*_M_	−13 dB	Efficiency of bias-Tee	*η*_1_	−1 dB
Gain/efficiency of first BPF	*G*_BPF1_/*η*_BPF1_	−5 dB	VCSEL saturation power	*P*_sat_	14.6 mW
Gain/efficiency of the second BPF	*G*_BPF2_/*η*_BPF2_	−12.84 dB	VCSEL threshold current	*I*_th_	2 mA
Power supply for LO	*P*_DC_	100 mW	VCSEL input bias voltage	*V*_bias_	2.7 V
Gain of the receiver	*G*_rec_	0 dB	VCSEL conversion efficiency	*η*	0.66
-	-	-	Fitness of the VCSEL conversion curve	*N*_*γ*_	2

#### SISO SNR

To evaluate the SNR of the two systems, specifically with the misalignment effect, two parameters matter: channel gain as the Tx/Rx is tilted or displaced, and transmitted power. As we found the similarity in the tradeoff between beam coverage and gain of THz antenna and optical lens, we try to guarantee the same beam coverage for the two systems to make a fair comparison in the following numerical analysis, i.e., HPBW of THz antennas at both Tx and Rx sides, half-power beam divergence (HPBD) of VCSEL beams, and FoV of the Rx in the VCSEL-based OWC system (see Eqs. (4) and (7) in the “Methods” section) are the same. The bandwidth *B* = 1 GHz is set for both systems.

**Fig. 2:**
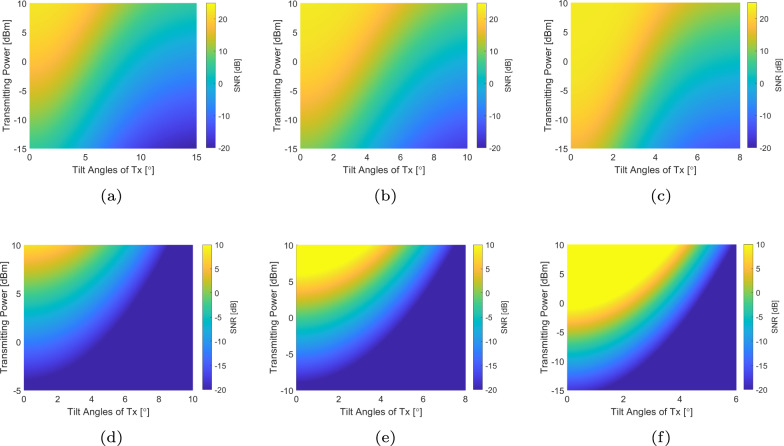
SNR comparison of TeraCom and VCSEL-based OWC systems with various transmitting power and tilt angles of Tx, while the HPBW of TeraCom and HPBD of VCSEL-based OWC are the same as 4°, 6°, and 8°. *θ*_CPC_ indicates the acceptance angle of the compound parabolic concentrator (CPCs) in VCSEL-based OWC systems. **a** THz, HPBW = 8°. **b** THz, HPBW = 6°. **c** THz, HPBW = 4°. **d** VCSEL, 2*θ*_CPC_ = HPBD = 8°. **e** VCSEL, 2*θ*_CPC_ = HPBD = 6°. **f** VCSEL, 2*θ*_CPC_ = HPBD = 4°.

As in Fig. 2, we evaluate the SNR of TeraCom and VCSEL-based OWC systems with various transmitting power *P*_t_ ranging from −15 dBm to 10 dBm and tilted angles of Tx ranging from 0^∘^ to 15^∘^, while HPBW = HPBD = 2*θ*_CPC_ = 8^∘^, 6^∘^, 4^∘^, where TeraCom antenna gains are approximately 28 dB, 31 dB, and 34 dB, respectively. From Fig. 2a–c, we can find that a lower HPBW which means that a more focused beam transmitted leads to a higher SNR with lower transmitting power; for example, as HPBW = 8^∘^, more than −10 dBm transmitting power is required to obtain >10 dB SNR if the transceivers are strictly aligned with each other, while no more than −15 dBm is needed as HPBW = 4^∘^. At the same time, it is evident that the system’s tolerance to misalignment effects has decreased. If we also take 10 dB SNR as a benchmark, the maximum allowed tilt angles with 10 dBm transmitting power degrade from around 8^∘^ to 4^∘^. As in Fig. 2d–f, we can find similar trends in required transmitting power and misalignment tolerance for VCSEL-based OWC systems. However, SNR obtained with the same configuration but with a larger HPBD is comparably lower, and the required power is higher. However, with a more focused VCSEL beam, i.e., HPBD = 4^∘^, the SNR remains consistently high throughout the system coverage area. From the comparison, two reasons leading to more required power in VCSEL-based OWC: (i) for OWC, the optical beam intensity needs to be converted into electrical current through photodetector devices, which leads to the power of channel gain when obtaining SNR in VCSEL-based OWC system as in Eq. (9); (ii) for OWC, non-constraint requirements need to be satisfied so that the signal power is generally lower than total transmitted beam power, i.e., relation between transmitting power and signal power is ${P}_{{\rm{sig}}}=\frac{1}{9}{P}_{{\rm{t}}}^{2}$ if direct-current optical orthogonal frequency division multiplexing (DCO-OFDM) is employed.

**Fig. 3:**
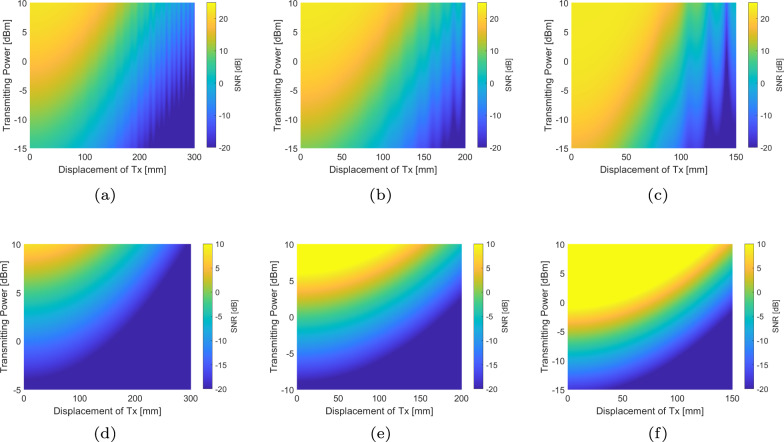
SNR comparison of TeraCom and VCSEL-based OWC systems with various transmitting power and displacement distance along x-axis of Tx, while the HPBW of TeraCom and HPBD of VCSEL-based OWC are the same as 4°, 6°, and 8°. **a** THz, HPBW = 8°. **b** THz, HPBW = 6°. **c** THz, HPBW = 4°. **d** VCSEL, 2*θ*_CPC_ = HPBD = 8°. **e** VCSEL, 2*θ*_CPC_ = HPBD = 6°. **f** VCSEL, 2*θ*_CPC_ = HPBD = 4°.

The same simulations are conducted for the comparison while Tx is displaced with a distance along the x-axis ranging from 0 to 400 mm, as in Fig. 3. We can find similar trends as in the case where the Tx is with tilt angles. The SNR results of VCSEL-based OWC systems, as shown in Fig. 3d–f, can be correlated to those with a tilted transmitter through geometric relationships. However, as in Fig. 3a–c, the TeraCom system appears to have a weaker tolerance to misalignment. This is because the displacement of the Tx is equivalent to that of both the Tx and Rx antennas having a tilt angle. Besides, we can see stripes in Fig. 3a–c, which are caused by the multi-path effect of THz wave propagation.

Although we plot the SNR for both systems with varying transmitting power, the maximum achievable and allowable transmitting power of the TeraCom and VCSEL-based OWC systems represent their respective limitations. For the TeraCom system, the achievable transmitting power from the transmitter is still limited at high frequencies. As can be found in the literature, the transmitting power at 350 GHz is still around 40 μW. However, for VCSEL-based OWC systems, laser beams can be generated with super high power, while the eye-safety regulations set the limits for the maximum allowed transmitting power. Considering the most conservative requirement with the original beam waist and optical lenses, the transmitting power is set at *P*_t_ = 1 mW.

**Fig. 4:**
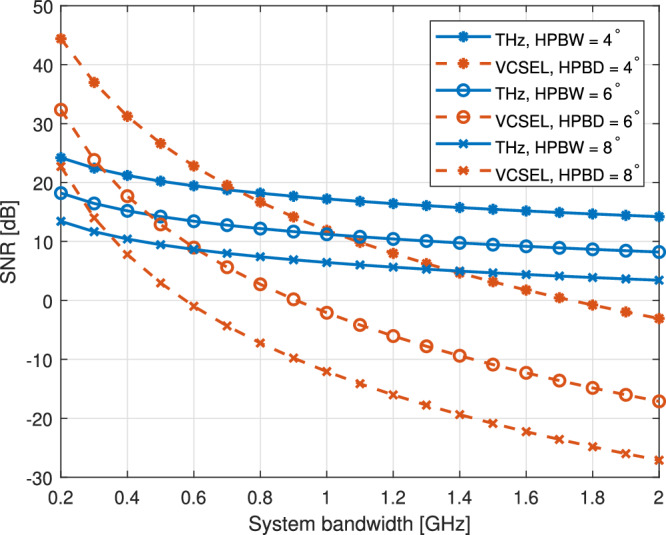
SNR comparison of TeraCom and VCSEL-based OWC systems with various system band widths, while HPBW of TeraCom and HPBD of VCSEL-based OWC are the same as 4°, 6°, and 8°.

As bandwidth is also a limitation of VCSEL-based OWC systems, we compare the SNR of the two systems with varying bandwidth as depicted in Fig. 4, which in VCSEL-based OWC systems is mainly restricted by the PD, and in TeraCom systems impacts system noises. In this simulation, the transmitting power for the VCSEL beam is set at ${P}_{{\rm{t}}}^{{\rm{v}}}=1\,{\rm{mW}}$, while the power for transmitting THz waves is ${P}_{{\rm{t}}}^{{\rm{thz}}}=40\mu$W. The results indicate a decline in SNR with increasing system bandwidth in both systems, with the VCSEL-based OWC system being more sensitive to changes in bandwidth. Additionally, we can see that reducing the HPBD by using optical lenses to create a more focused beam can effectively address this issue, allowing for higher bandwidth while maintaining the required SNR. Still, it’s challenging for VCSEL-based OWC to outperform the TeraCom systems if the bandwidth exceeds 1 GHz with the current receiver design. Meanwhile, numerous studies, demonstrations, and products focused on PD design with higher bandwidth have emerged to address this issue.

#### SISO CF

**Fig. 5:**
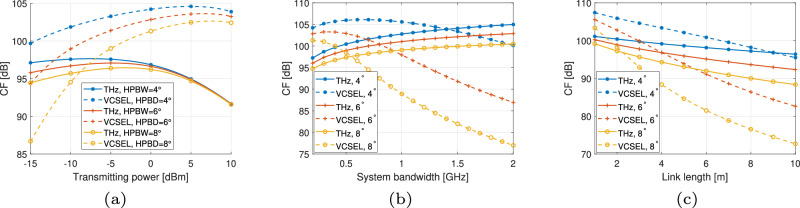
CF comparison of TeraCom and VCSEL-based OWC systems under varying transmitting power, system bandwidth, and link length. **a** *B* = 500 MHz, *L* = 2 m. **b** *L* = 2 m. **c** *B* = 500 MHz.

Energy efficiency is a crucial metric for next-generation networks. First, we compare the CF of the two systems under varying transmitting power, system bandwidth, and link length to analyze the factors influencing system performance, assuming Tx and Rx are strictly aligned with each other, as in Fig. 5. Still, we standardize the HPBW of antenna patterns at both the Tx and Rx sides in the TeraCom system, the HPBD of VCSEL beams, and the FoV of VCSEL-based OWC receivers to evaluate system performance under the same conditions for handling misalignment. Figure 5a depicts the CF of the two systems as a function of transmitting power. As the beam-focusing ability, i.e., beam energy concentration, increases, the channel gain improves, enhancing CF performance for both systems. However, beam energy concentration matters more in VCSEL-based OWC systems. Higher beam divergence requires higher transmitting power to ensure the system’s performance. Additionally, as the transmitting power increases, the CF for both systems initially rises and then declines. In VCSEL-based OWC systems, the saturation of electro-optic conversion in the VCSEL transmitter contributes to this decline. In contrast, in TeraCom systems, the dominance of DC power consumed by cascaded components at the transceivers leads to a decrease. We use the maximum achievable and allowable transmitting power for both systems to analyze the impacts of bandwidth and link length, respectively. In Fig. 5b, the CF of the VCSEL-based OWC system initially increases and then decreases due to the tradeoff between bandwidth and receiving area of the PD at the Rx, which limits the receiving power. At much higher bandwidths, the limitations on receiving power become more significant for CF than the contributions of bandwidth to the data rate. In contrast, for TeraCom systems, although an increase in bandwidth leads to higher noise levels, the contribution of bandwidth to the data rate is dominant in determining CF. However, enhancing the energy concentration of VCSEL beams will significantly help mitigate PD bandwidth limitations. In evaluations of link length impact on CF, we only consider the LoS components in THz channels for simplicity. As in Fig. 5c, performance degradation occurs in both systems as link lengths increase, with the VCSEL-based OWC exhibiting greater sensitivity. While beam focusing improves performance, the VCSEL-based OWC is more suitable for short-range scenarios due to its higher beam divergence, which exacerbates distance-dependent losses.

**Fig. 6:**
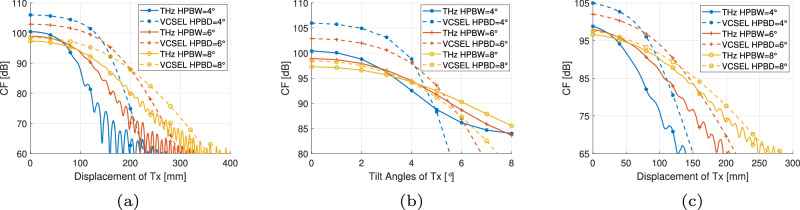
CF comparison of TeraCom and VCSEL-based OWC systems with misalignment between Tx and Rx. **a** Tilt angle 0°. **b** Displacement 0 mm. **c** Tilt angle 2°.

By integrating the performance evaluation models proposed for numerical analysis under misalignment conditions, we further illustrate the CF comparison when the Tx is tilted, displaced, or when both tilting and displacement are present, as in Fig. 6. The results are obtained under the configuration that bandwidth *B* = 500 MHz and link length *L* = 2 m for both systems, ${P}_{{\rm{t}}}^{{\rm{thz}}}=40\mu$W for TeraCom system, and ${P}_{{\rm{t}}}^{{\rm{v}}}=1$ mW for VCSEL-based OWC system. As in Fig. 6a, b, VCSEL-based OWC outperforms TeraCom in terms of CF under the same beam coverage conditions, and this advantage becomes more pronounced with increasingly focused beams. In Fig. 6c, we show CF as a function of Tx displacement distance with a tilt angle of 2^∘^ at the same time. In conclusion, the impact of misalignment, specifically tilting and displacement, on VCSEL-based OWC performance arises from diffraction loss due to the shift between the beam area and the receiving area on the Rx side. In contrast, for TeraCom systems, the radiation patterns from both the Tx and Rx sides, contributing to the overall antenna gain in a specific LoS direction, determine system performance under misalignment conditions. Moreover, Figs. 5 and 6 demonstrate that under unified focusing ability, VCSEL-based OWC exhibits significantly superior energy efficiency compared to TeraCom systems. Despite normalizing the alignment effects, the cascaded transceiver architecture in THz systems inherently introduces compounded power inefficiency. However, the VCSEL-based system maintains high energy efficiency even when the receiver bandwidth is substantially reduced to ensure fair beam coverage comparison. This highlights VCSEL’s inherent advantage in low-power operation, whereas THz systems require complex, power-intensive components to compensate for alignment-induced performance degradation.

#### Coverage probability

**Fig. 7:**
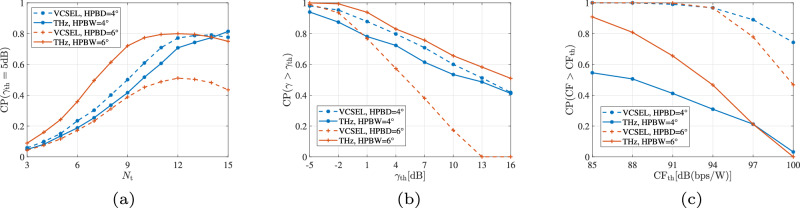
Coverage probability in terms of SNR and CF. **a** SNR threshold 5 dB. **b** *N*_t_ = 12. **c** *N*_t_ = 12.

To verify the coverage probability of the two technologies with multiple transmitters mounted on the ceiling of the room, we analyze the following 3 cases in Fig. 7: (i) probability of the signal-to-interference-and-noise ratio (SINR) by a single user at arbitrary locations inside the room is larger than threshold of 5 dB; (ii) probability of SINR with varying threshold as *N*_t_ = 15 where *N*_t_ × *N*_t_ gives the total transmitter number; and (iii) probability of CF with varying threshold as *N*_t_ = 15. The configurations are set as bandwidth *B* = 500 MHz and link length *L* = 2 m for both systems, ${P}_{{\rm{t}}}^{{\rm{thz}}}=40\mu$W for the TeraCom system, and ${P}_{{\rm{t}}}^{{\rm{v}}}=1$ mW for the VCSEL-based OWC system. Figure 7a shows that as *N*_t_ increases, coverage probability (CP) for both systems initially improves, reaching an optimal point before degrading due to interference noise when HPBW = HPBD = 6^∘^. Overall, the CP for the TeraCom system is higher than that of the VCSEL-based OWC system. When HPBW = HPBD = 4^∘^, CP increases as *N*_t_ improves from 3 to 15 for the TeraCom system, while in VCSEL-based OWC systems, there is still a saturation point. However, the VCSEL-based OWC system outperforms the TeraCom system, highlighting the importance of using a focused beam for optimal performance in VCSEL-based OWC systems.

From Fig. 7a, we choose *N*_t_ = 12 to validate the coverage probability with varying thresholds in terms of SINR and CF, as in Fig. 7b, c, respectively. In Fig. 7b, both systems exhibit limited performance in a networked configuration, underscoring the critical need for beamforming or beam steering in this context. Still, beam focusing significantly impacts system performance regarding SINR, with the VCSEL-based OWC system being more dependent on it. At higher SINR thresholds, the VCSEL-based OWC cannot outperform TeraCom. However, as shown in Fig. 7c, the VCSEL-based OWC consistently outperforms in terms of CF, which is calculated solely based on the serving transmitter’s power consumption. Besides, for TeraCom, a more focused beam does not necessarily lead to improved system performance.

### Numerical comparison in an outdoor environment

This subsection shows the FSO and THz link performance in outdoor environments and especially in UAV applications with exemplary system parameters. FSO link at 1550 nm wavelength and THz link at 350 GHz are chosen. The 1550 nm wavelength is adopted for FSO links due to its dual advantage of low atmospheric attenuation and compatibility with mature fiber-optic components^2^. For numerical analysis with stochastic models (see “Methods” section), the commonly used parameters for the FSO link are *ρ* = 0.596, *Ω* = 1.3265, *b*_0_ = 0.1079, and for the THz link are *a* = 80, *α* = 3, *μ* = 1, *b*_0_ = 0.1079, $\hat{{g}_{{\rm{t}}}}=1$. The weather-dependent parameters for the FSO link are from ref. ^47^. For the stochastic channel models considered, we adopt analytical expressions that were previously derived and validated by Monte Carlo simulations in the corresponding literature^47,48^. Therefore, the channel behavior is directly modeled in MATLAB using these analytical expressions, without repeating stochastic simulations.

#### Impact of environmental conditions

**Fig. 8:**
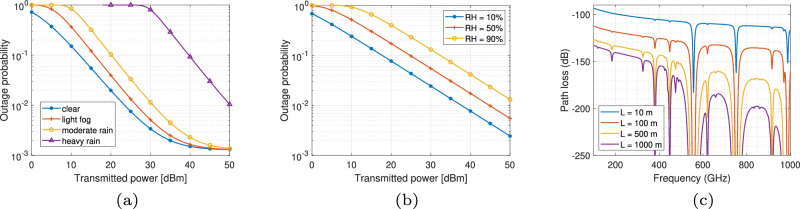
FSO and THz links with various weather conditions, humidity, and frequency-dependent THz path loss. **a** FSO under various weather. **b** THz under varying humidity. **c** THz spectral window.

Figure 8 illustrates the performance of FSO and THz communication links under various environmental conditions and path loss characteristics. Here, the outage probability indicates the probability that the instantaneous SNR falls below 5 dB. Model (41) is used for the THz link. For both links, original beam waist *ω*_0_ = 3 m, link length *L* = 1 km, total displacement square variance *σ*_m_ = 1.5 m, radius of receiving plane 5 cm, and noise power of −10 dBm are set^48^. For the FSO link, FoV of the receiver *θ*_FoV_ = 20 mrad, the responsivity equals 0.7, and clear weather condition is assumed. For the THz link, antenna gains at both transceivers are 55 dBi and relative humidity (RH) = 10%.

As in Fig. 8a, the outage probability as a function of transmitted power for FSO links under different weather scenarios, including clear weather, light fog, moderate rain, and heavy rain, is depicted. As expected, adverse weather conditions (e.g., rain and fog) result in higher outage probabilities at lower transmitting powers, with heavy rain having the most severe impact on signal reliability. Figure 8b presents the outage probability for THz communication links with varying relative humidity levels (RH = 10%, 50%, and 90%). As the transmitted power increases, the outage probability decreases, but higher humidity levels (e.g., 90% RH) cause significantly higher outage probabilities, indicating that THz link performance is sensitive to atmospheric humidity. Figure 8c further depicts path loss in dB across a range of frequencies (0–1000 GHz) for different transmission distances (*L* = 10 m, 100 m, 500 m, and 1000 m). The graph reveals spectral windows where path loss is minimized, interspersed with frequencies experiencing high path loss due to molecular absorption loss (MAL). Longer distances lead to greater path loss and narrow the effective spectral window, emphasizing the importance of frequency selection and distance in THz link design. Given the differing performance of FSO and THz links under various environmental conditions, a hybrid link design could effectively compensate for each of their limitations.

#### Comparison of two THz link models

**Fig. 9:**
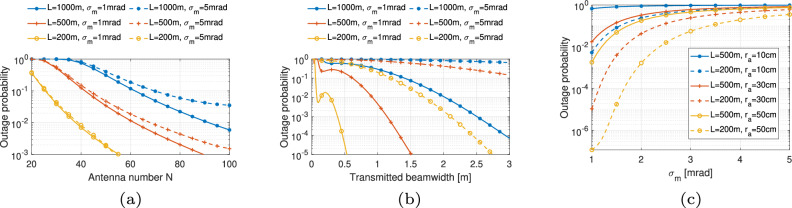
Comparison of THz link with models (41) and (45). **a** THz model (45) with *N*_ant_. **b** THz model (41) with ω_0_. **c** THz model (41) with σ_m_.

Figure 9 presents a comparison of THz link performance using two models, Model (45) and Model (41), considering the effects of antenna number, transmitted beamwidth, and pointing error. In this analysis, transmitted power is fixed at 5 dBm with noise power of 69 dBm, and RH = 10%. Figure 9a shows the outage probability as a function of the number of antennas *N*_ant_, with different transmission distances *L* (1000 m, 500 m, and 200 m) and pointing error standard deviations *σ*_m_ (1 mrad and 5 mrad). In model (45), the number of antennas determines the link gain and radiation HPBW, which influences the system’s resilience to pointing errors. A higher number of antennas generally reduces outage probability, with shorter distances and smaller pointing errors further improving link reliability. On the contrary, in model (41), the impact of pointing error on THz system performance is determined by the receiving beam radius, similar to the model for the FSO link, as shown in Fig. 9b. The received beam radius depends on the transmitted beam radius according to the divergence principle of electromagnetic wave propagation. At the same time, a larger transmitted beam radius is aligned with the narrower HPBW, leading to higher antenna gain. Thus, a larger beam radius with higher gain reduces the outage probability, especially at shorter distances. Figure 9c examines the impact of pointing error on outage probability for different receiver antenna radii *r*_a_ (10 cm, 30 cm, and 50 cm) and distances *L*, where *ω*_0_ = 0.1 m. As the pointing error standard deviation *σ*_m_ increases, the outage probability rises, particularly for smaller receiver antenna radii and longer distances. Larger receiver antenna radii improve the system’s resilience to pointing errors, enhancing reliability over extended distances.

By comparing the two models, we found that model (45) is less sensitive to link length. However, effective antenna area will also have an impact on the system performance, considering the beam divergence, which is not demonstrated in model (45). Hence, the combination of two models to showcase full beam propagation characteristics is worthy of further investigation.

#### Performance in UAV applications

**Fig. 10:**
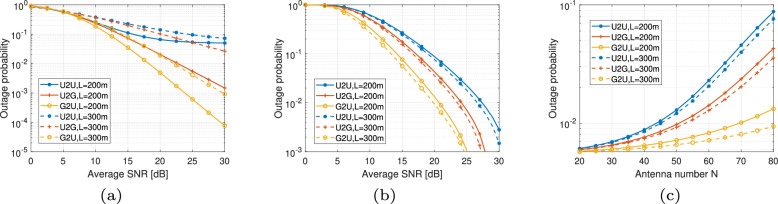
Performance of FSO and THz links in UAV applications. **a** FSO. **b** THz. **c** THz antenna number impact.

Figure 10 illustrates the performance of FSO and THz links in UAV applications, showing the outage probability for different link types, i.e., UAV-to-UAV (U2U), UAV-to-Ground (U2G), Ground-to-UAV (G2U), over varying distances *L* (200 m and 300 m). We use model (45) to present the performance of THz links, where for FSO links, *ω*_0_ = 3 m, *r*_*a*_ = 5 cm, *θ*_FOV_ = 20 mrad, and light fog weather condition is assumed. Correspondingly, RH = 40% and *N*_ant_ = 80 are set for the THz link. Primary parameters of misalignment for different links are from ref. ^47^. Figure 10a shows the outage probability versus average SNR in dB for FSO links. Results indicate that G2U links have the lowest outage probability, followed by U2G links, and then U2U links. As the distance *L* increases from 200 m to 300 m, outage probability increases across all link types due to higher path loss at longer distances. Similar to the FSO case, Fig. 10b presents the outage probability as a function of average SNR for THz links. The same trend is observed where G2U links show superior performance, followed by U2G and U2U links. Higher average SNR levels lead to significantly reduced outage probability, especially for shorter distances (200 m) compared to longer distances (300 m). Through the comparison, we can find that the FSO link is more sensitive to link length, and for the U2U link with the largest pointing error probabilities, THz communications perform better. Besides, Fig. 10a, b demonstrates G2U’s superior performance over U2G, which directly results from the asymmetric error propagation in Eq. (47): ground transmitters eliminate the distance-squared error amplification (*L*^2^*σ*^4^) inherent to UAV-originated links. Figure 10c examines the effect of the number of antennas *N*_ant_ on the outage probability for THz links. As the average SNR is fixed at 20 dB, increasing the antenna number primarily demonstrates the effect of a narrower beamwidth, which increases the system’s sensitivity to pointing errors.

## Discussion

This study provides a quantitative assessment of THz and OWC technologies across indoor and outdoor environments, revealing key strengths and limitations of each. For indoor settings, our analysis shows that VCSEL-based OWC has higher energy efficiency than TeraCom but is more susceptible to distance and bandwidth limitations, requiring more focused beam transmission to address these challenges. In outdoor scenarios, stochastic channel models reveal that weather, absorption, and pointing errors significantly impact both FSO and THz links. FSO, with its heightened sensitivity to transmission distance and alignment, is less suitable for UAV-to-UAV applications compared to THz links. These findings underscore the importance of tailored technology choices depending on specific application needs and environmental conditions, providing guidance for selecting the optimal technology in both stable and dynamic environments.

Additionally, several important aspects that could affect the modeling and comparison results deserve further attention, including:

- We note that the quantitative comparison results presented in this study are inherently sensitive to the adopted channel models. While we adopt representative multi-ray and stochastic models that capture key propagation phenomena such as misalignment and environmental impairments, alternative modeling assumptions, such as turbulence models for optical links or higher-fidelity UAV motion dynamics, could slightly shift the absolute performance metrics. However, the core comparative trends observed between THz and optical wireless systems, particularly regarding their sensitivity to misalignment and environmental effects, are expected to remain robust across different reasonable model selections. Future work could further explore sensitivity analyses under more diverse channel conditions to consolidate the generality of the presented insights.
- In this work, we focused on a comparative analysis under conventional far-field assumptions, which are generally valid for most current THz and optical wireless communication scenarios. However, as future systems push towards shorter link distances, higher frequencies, and more compact device deployments, near-field effects may become increasingly significant. This shift necessitates the extension of existing channel models to capture wavefront curvature, non-uniform phase distributions, and spatial beam evolution in the near-field region. Incorporating near-field modeling would provide a more accurate characterization of emerging THz and VLC systems, particularly for ultra-dense networks, indoor hotspots, and short-range UAV communications, and is an important direction for future work.
- Apart from channel modeling uncertainties, hardware-level variations, such as fluctuations in transmitter power, receiver sensitivity, or beam divergence angles, can also impact practical system performance. As THz and OWC hardware technologies continue to evolve, these non-idealities could introduce additional deviations from theoretical predictions. Future studies could further investigate the joint effects of channel and hardware uncertainties to provide more comprehensive guidelines for system design and deployment.
- Finally, for UAV-specific scenarios, additional mobility-induced effects deserve further investigation. Beyond the static or quasi-static assumptions adopted in this study, UAV-to-UAV links may experience non-negligible delay spread, Doppler shifts, beam squint, and beam-split effects, particularly under high mobility or agile maneuvering conditions. These phenomena can influence synchronization accuracy, spectral efficiency, and beam tracking stability, thereby impacting the overall system. Incorporating such mobility-aware models represents an important avenue for future research, especially for dynamic airborne networks and next-generation UAV communication deployments.

## Methods

### Indoor applications: TeraCom vs. VCSEL-based OWC

This section introduces a typical VCSEL-based OWC system for indoor scenarios to compare with the TeraCom system. The channel gain models of the two systems for SISO links are presented first. After specifying the noises considered in the systems, e.g., phase noises in TeraCom, the SNR of the two systems is formulated. Besides, with typical system designs, the consumption factor is adopted to indicate the system’s energy efficiency. Based on models built for SISO links, distributed AP-based networks are considered to analyze the system performance on data rate as well as energy efficiency.

#### Channel model with misalignment of VCSEL-based OWC

For laser systems, the Gaussian beam propagation principle is widely adopted to express diffraction loss while the laser propagates in free space. In ref. ^36^, a Gaussian beam propagation-based unified misalignment channel model is built for VCSEL-based OWC, where the displacement error along *x* and *y-*axis, denoted by *x*_dis_ and *y*_dis_, azimuth and elevation angles of orientation error at the Tx and Rx side, denoted by ${\phi }_{{\rm{a}}}^{{\rm{t}}}$, ${\phi }_{{\rm{e}}}^{{\rm{t}}}$, ${\phi }_{{\rm{a}}}^{{\rm{r}}}$, and ${\phi }_{{\rm{e}}}^{{\rm{r}}}$ are included. The channel gain is expressed as follows^36^

1 $${H}_{{\rm{owc}}}={\iint }_{(x,y)\in {\mathcal{A}}}\frac{2}{\pi {w}^{2}(L)}\exp \left(-\frac{2{\rho }^{2}(x,y)}{{w}^{2}(L)}\right){\rm{d}}x{\rm{d}}y,$$

where ${\mathcal{A}}$ is the receiving PD surface. *w*(*L*) is the beam waist at *z* = *L* and *w*^2^(*L*) is defined by

2 $$\left\{\begin{array}{ll}{w}^{2}(L)={w}_{0}^{2}\left[1+{z}_{{\rm{R}}}^{-2}{({c}_{1}+x{c}_{2}+y{c}_{3}-{c}_{4})}^{2}\right]\quad \\ {c}_{1}:=L\cos {\phi }_{{\rm{e}}}^{{\rm{t}}}\cos {\phi }_{{\rm{a}}}^{{\rm{t}}},\quad {c}_{2}:=\cos {\phi }_{{\rm{e}}}^{{\rm{t}}}\sin \left({\phi }_{{\rm{a}}}^{{\rm{t}}}-{\phi }_{{\rm{a}}}^{{\rm{r}}}\right)\quad \\ {c}_{3}:=\sin {\phi }_{{\rm{e}}}^{{\rm{r}}}\cos {\phi }_{{\rm{e}}}^{{\rm{t}}}\cos \left({\phi }_{{\rm{a}}}^{{\rm{t}}}-{\phi }_{{\rm{a}}}^{{\rm{r}}}\right)+\cos {\phi }_{{\rm{e}}}^{{\rm{r}}}\sin {\phi }_{{\rm{e}}}^{{\rm{t}}}\quad \\ {c}_{4}:={x}_{{\rm{dis}}}\cos {\phi }_{{\rm{e}}}^{{\rm{t}}}\sin {\phi }_{{\rm{a}}}^{{\rm{t}}}-{y}_{{\rm{dis}}}\sin {\phi }_{{\rm{e}}}^{{\rm{t}}}\quad \end{array}\right.,$$

where *w*_0_ is the primary beam waist radius, and ${z}_{{\rm{R}}}=\pi {w}_{0}^{2}/\lambda$ with *λ* indicating the beam wavelength. Besides, *ρ*(*x*, *y*) is the Euclidean distance of the point (*x*, *y*) from the center of the beam spot, and *ρ*^2^(*x*, *y*) is expressed as

3 $$\begin{array}{ll}{\rho }^{2}(x,y)\,=\,{\left[L-x\sin {\phi }_{{\rm{a}}}^{{\rm{r}}}+y\cos {\phi }_{{\rm{a}}}^{{\rm{r}}}\sin {\phi }_{{\rm{e}}}^{{\rm{r}}}\right]}^{2}\\\qquad\qquad+{\left[x\cos {\phi }_{{\rm{a}}}^{{\rm{r}}}+y\sin {\phi }_{{\rm{a}}}^{{\rm{r}}}\sin {\phi }_{{\rm{e}}}^{{\rm{r}}}-{x}_{{\rm{dis}}}\right]}^{2}\\\qquad\qquad\,+{(y\cos {\phi }_{{\rm{e}}}^{{\rm{r}}}-{y}_{{\rm{dis}}})}^{2}+{({c}_{1}+x{c}_{2}+y{c}_{3}-{c}_{4})}^{2}.\end{array}$$

#### Channel gain enhancement of VCSEL-based OWC

The channel gain is closely related to the primary beam waist radius *w*_0_. That is, smaller *w*_0_ leads to larger beam divergence, and thus larger transmission loss. Generally, an optical element such as a lens is embedded in front of the VCSEL transmitter to enlarge the beam waist, achieving beam focusing to enhance the received signal power. Denoting *G*_lens_ as the magnification factor of the lens, which is defined as the ratio of the transformed beam waist to the original beam waist, the transformed primary beam waist ${w}_{0}^{{\prime} }={G}_{{\rm{lens}}}{w}_{0}$. There is also a tradeoff between the magnification factor and beam divergence. Adopting the HPBD full angle as a metric, this tradeoff is formulated as^49^

4 $${\theta }^{{\rm{Mag}}}=\sqrt{2\ln 2}\frac{\lambda }{\pi {G}_{{\rm{lens}}}{w}_{0}}.$$

*θ*^Mag^ is the HPBD full angle after the lens from the VCSEL-based system.

Besides, the PD area impacts the channel gain. However, there is a tradeoff between the bandwidth and area of a PD, which is expressed as

5 $$B={\left(\sqrt{\frac{4\pi {\varepsilon }_{0}{\varepsilon }_{{\rm{r}}}{R}_{{\rm{L}}}}{0.44{v}_{{\rm{s}}}}{A}_{{\rm{PD}}}}\right)}^{-1},$$

where *A*_PD_ indicates the PD area and *R*_L_ is the load resistance of the detector. Permittivity in vacuum *ε*_0_ = 8.854 × 10^12^ F/m, relative permittivity of the semiconductor *ε*_r_ = 12, carrier saturation velocity *v*_s_ = 1 × 10^5^ m/s are taken from ref. ^50^. One of the existing approaches utilizes CPCs to increase the effective area of a PD as^45^

6 $${\hat{A}}_{{\rm{PD}}}=\frac{\pi }{4}{G}_{{\rm{CPC}}}{A}_{{\rm{PD}}},$$

where *G*_CPC_ is the CPC gain limited by the acceptance angle *θ*_CPC_ for the incident beam as

7 $${G}_{{\rm{CPC}}}=\frac{1}{{\sin }^{2}{\theta }_{{\rm{CPC}}}}.$$

#### Signal-to-noise ratio of VCSEL-based OWC

**Fig. 11:**
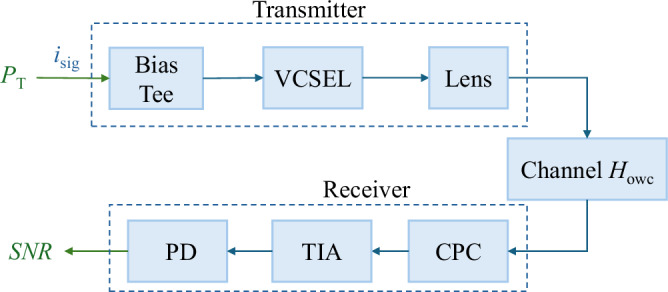
Block diagram of VCSEL-based OWC transceiver architecture.

A typical VCSEL-based OWC system architecture contains a transmitter and a receiver, as depicted in Fig. 11. At the transmitter, a bias-Tee supplying power for the VCSEL, a VCSEL, and optical elements to focus the beam are included; while at the receiver, the detector, i.e., PD, and trans-impedance amplifier (TIA) are contained. To formulate the SNR of the VCSEL-based OWC systems, shot noise, thermal noise, and laser relative intensity noise are considered. The noise variance of the VCSEL-based OWC is given by

8 $${\sigma }^{2}=\frac{4kTB{F}_{{\rm{n}}}}{{R}_{{\rm{L}}}}+2qB{R}_{{\rm{PD}}}{H}_{{\rm{owc}}}{P}_{{\rm{t}}}+B{N}_{{\rm{RI}}}{({R}_{{\rm{PD}}}{H}_{{\rm{owc}}}{P}_{{\rm{t}}})}^{2},$$

where *q* is the elementary charge, *B* is the system bandwidth, *R*_PD_ is the responsivity of PD, *P*_t_ is the transmitted laser beam power from VCSEL, *k* is Boltzmann constant, *T* is the temperature in Kelvin, *F*_n_ is the noise figure of the TIA, and *N*_RI_ is the power spectral density of the VCSEL’s relative intensity noise defined as the mean square of instantaneous power fluctuations divided by the squared average power of the laser source.

Meanwhile, the optical direct-current (DC) bias power is required to comply with the non-negativity constraint and guarantee better communication performance. For the DCO-OFDM scheme, the average signal power ${P}_{{\rm{sig}}}=\frac{1}{9}{P}_{{\rm{t}}}^{2}$ is chosen to discard the clipping noise effectively. Then, the SNR of the VCSEL-based OWC system is derived as

9 $${\gamma }_{{\rm{owc}}}=\frac{{R}_{{\rm{PD}}}^{2}{H}_{{\rm{owc}}}^{2}{P}_{{\rm{sig}}}}{{\sigma }^{2}}.$$

#### Consumption factor of VCSEL-based OWC

At the VCSEL-based OWC transmitter, DC power and signal are input into a bias-Tee and then injected into the VCSEL to be converted to beam power. The photoelectric conversion is non-linear. Light intensity-current-voltage (LIV) curves are used to represent the input-output characteristics of VCSELS, which can be obtained through measurements. In^35^, the relationship between the output optical power *P*_out_ and the input drive current *I*_in_ of the VCSEL is

10 $${P}_{{\rm{out}}}=\left\{\begin{array}{ll}\frac{\eta ({I}_{{\rm{in}}}-{I}_{{\rm{th}}})}{{\left[1+{\left(\frac{\eta ({I}_{{\rm{in}}}-{I}_{{\rm{th}}})}{{P}_{{\rm{sat}}}}\right)}^{2{N}_{\gamma }}\right]}^{\frac{1}{2{N}_{\gamma }}}},\quad &{I}_{{\rm{in}}}\ge {I}_{{\rm{th}}}\\ 0,\quad &{I}_{{\rm{in}}} < {I}_{{\rm{th}}}\end{array}\right.,$$

where *P*_sat_ is the saturation power, *I*_th_ is the threshold current, *η* is the conversion efficiency in W/A, and *N*_*γ*_ is a parameter describing the curve fineness^35^. These parameters are obtained through measurement and data fitting. We denote *η*_2_( ⋅ ) as an operator to indicate the non-linear function in Eq. (10). Given input bias voltage *V*_bias_, power injected to the VCSEL is represented by ${\eta }_{2}^{-1}({P}_{{\rm{t}}})$, where ${\eta }_{2}^{-1}$ is the operation for the inverse function. Assume the efficiency of bias-Tee is *η*_1_, the power required at the transmitter, including DC and signal power, is expressed as

11 $${P}_{{\rm{T}}}=\frac{{\eta }_{2}^{-1}({P}_{{\rm{t}}})}{{\eta }_{1}}.$$

If the power consumed by signal processing, analog-to-digital converter (ADC), and digital-to-analog converter (DAC) processes is altogether denoted by *P*_others_, and DC power may be consumed at the receiver side by TIA is denoted by *P*_R_, the CF of the VCSEL-based OWC system is

12 $${{\rm{CF}}}_{{\rm{owc}}}=\frac{B\,{\log }_{2}(1+{\gamma }_{{\rm{owc}}})}{{P}_{{\rm{T}}}+{P}_{{\rm{R}}}+{P}_{{\rm{others}}}}.$$

#### Channel gain model of TeraCom

We adopt a multi-ray channel model for TeraCom, which utilizes the principles of geometric optics to trace the propagation of line-of-sight (LoS), reflected, diffusely scattered, and diffracted electromagnetic (EM) waves. Assume there are *N*_Ref_ reflected rays, *N*_Sca_ scattered rays, *N*_Dif_ diffracted rays, the transfer function of the channel at frequency *f* is^41^

13 $$H(f)={H}_{{\rm{LoS}}}(f)+\mathop{\sum }\limits_{p=1}^{{N}_{{\rm{Ref}}}}{H}_{{\rm{Ref}}}^{(p)}(f)+\mathop{\sum }\limits_{q=1}^{{N}_{{\rm{Sca}}}}{H}_{{\rm{Sca}}}^{(q)}(f)+\mathop{\sum }\limits_{u=1}^{{N}_{{\rm{Dif}}}}{H}_{{\rm{Dif}}}^{(u)}(f),$$

where ${H}_{{\rm{LoS}}}(f),{H}_{{\rm{Ref}}}^{(p)}(f),{H}_{{\rm{Sca}}}^{(q)}(f)$, and ${H}_{{\rm{Dif}}}^{(u)}(f)$ are transfer functions for the LoS, reflected, scattered, and diffracted propagation paths, respectively, which will be specified below.

*H*_LoS_ relates to the antenna gain, spreading loss (SL), and MAL, which is expressed by

14 $$\begin{array}{rcl}{H}_{{\rm{LoS}}}(f)&=&{A}_{{\rm{LoS}}}{H}_{{\rm{SL}}}(f){H}_{{\rm{MAL}}}(f){{\rm{e}}}^{-j2\pi f{\tau }_{{\rm{LoS}}}}\\ &=&\sqrt{{G}_{{\rm{LoS}}}^{{\rm{Tx}}}{G}_{{\rm{LoS}}}^{{\rm{Rx}}}}\frac{c}{4\pi fr}{{\rm{e}}}^{\frac{1}{2}\kappa (f)r}{{\rm{e}}}^{-j2\pi f{\tau }_{{\rm{LoS}}}}\end{array}$$

where *r* is the LoS distance between transmitter and receiver, and time delay *τ*_LoS_ = *r*/*c*. *κ*(*f*) is a frequency-dependent medium absorption coefficient modeled specifically for the 100–450 GHz frequency band in ref. ^37^. ${G}_{{\rm{LoS}}}^{{\rm{Tx}}}$ and ${G}_{{\rm{LoS}}}^{{\rm{Rx}}}$ are angular-dependent antenna gain patterns at transmitter and receiver, respectively, which can be simplified by a Gaussian beam model as^51^

15 $$G({\theta }_{{\rm{a}}},{\theta }_{{\rm{e}}})={G}_{0}\cdot {{\rm{e}}}^{-{\left(\frac{{\theta }_{{\rm{a}}}-{\theta }_{{\rm{a}}}^{0}}{{\theta }_{{\rm{a}}}^{{\rm{bw}}}}\right)}^{2}}\cdot {{\rm{e}}}^{-{\left(\frac{{\theta }_{{\rm{e}}}-{\theta }_{{\rm{e}}}^{0}}{{\theta }_{{\rm{e}}}^{{\rm{bw}}}}\right)}^{2}},$$

where ${\theta }_{{\rm{a}}}^{{\rm{bw}}}$ and ${\theta }_{{\rm{e}}}^{{\rm{bw}}}$ describe the HPBW of the radiated pattern in the azimuth and elevation, ${\theta }_{{\rm{a}}}^{0}$ and ${\theta }_{{\rm{e}}}^{0}$ are the main beam directions. *θ*_a_, *θ*_e_ are the azimuth and elevation angles from the source point to the target point, and *G*_0_ is the maximum gain. Eq. (15) is experimentally validated for horn antennas, where the relation between ${\theta }_{{\rm{a,e}}}^{{\rm{bw}}}$ and *G*_0_ is obtained approximately, assuming a square antenna aperture as

16 $${\theta }_{{\rm{a,e}}}^{{\rm{bw}}}=2\sqrt{\frac{\pi }{{G}_{0}}}.$$

Transfer functions for reflected, scattered, and diffracted ray propagation are given as follows:

17 $$\begin{array}{rcl}{H}_{{\rm{Ref}}}(f)&=&\frac{{A}_{{\rm{Ref}}}R(f)c}{4\pi f({r}_{1}+{r}_{2})}{{\rm{e}}}^{-j2\pi {\tau }_{{\rm{Ref}}}-\frac{1}{2}\kappa (f)({r}_{1}+{r}_{2})}\\ {H}_{{\rm{Sca}}}(f)&=&\frac{{A}_{{\rm{Sca}}}S(f)c}{4\pi f({s}_{1}+{s}_{2})}{{\rm{e}}}^{-j2\pi {\tau }_{{\rm{Sca}}}-\frac{1}{2}\kappa (f)({s}_{1}+{s}_{2})}\\ {H}_{{\rm{Dif}}}(f)&=&\frac{{A}_{{\rm{Dif}}}L(f)c}{4\pi f({d}_{1}+{d}_{2})}{{\rm{e}}}^{-j2\pi {\tau }_{{\rm{Dif}}}-\frac{1}{2}\kappa (f)({d}_{1}+{d}_{2})}\end{array},$$

where *A*_Ref_, *A*_Sca_, and *A*_Dif_ denote the antenna gains in reflected, scattered, and diffracted ray propagation, respectively. {*r*/*s*/*d*}_1_ and {*r*/*s*/*d*}_2_ represent the distance from the transmitter to the reflecting/scattering/diffracting point and the distance from these points to the receiver. *τ*_Ref_ = *τ*_LoS_ + (*r*_1_ + *r*_2_ − *r*)/*c*, and *τ*_Sca_, *τ*_Dif_ are calculated in the same manner. Besides, *R*(*f*), *S*(*f*), *L*(*f*) denote reflection, scattering, and diffraction coefficients for a rough surface, of which the details can be found in ref. ^41^. Given a room geometry and transmitter/receiver locations, reflected point locations are calculated. Then, scatterers are assumed as a set of points surrounding reflecting points. In indoor environments, diffraction effects are negligible as the region is close to the incident shadow boundary^41^. Finally, the channel gain for TeraCom is *H*_thz_ = ∣*H*(*f*)∣^2^.

#### Signal-to-noise ratio of TeraCom

**Fig. 12:**
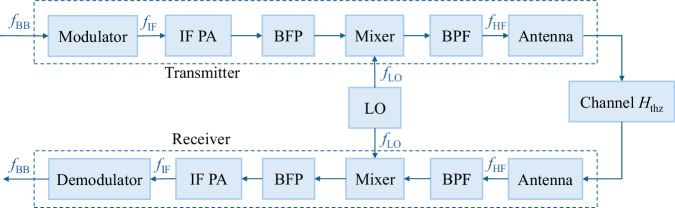
Block diagram of electronics-based TeraCom transceiver architecture.

We take an electronics-based TeraCom system as an example, which generally has a symmetrical transceiver design, as shown in Fig. 12. At the transmitter, the baseband signal is modulated to an intermediate frequency (IF) by the modulator at first, and then amplified by an IF power amplifier (PA) and mixed with the local oscillator (LO) signal by a mixer. After the bandpass filter (BPF), the THz signal is transmitted by an antenna. At the receiver, captured by the antenna, the downconversion is applied to THz waves, which is exactly the reverse process of that in the transmitter. Assuming an additive white Gaussian noise (AWGN) channel, the received SNR at the receiver is defined as

18 $${\gamma }_{0}=\frac{{P}_{{\rm{rec}}}}{{\sigma }_{w}^{2}}=\frac{{P}_{{\rm{rec}}}}{kTBF},$$

where *P*_rec_ is the received signal power and *F* is the noise figure at the receiver. However, the phase noise (PN) of local oscillators (LO) is negligible in TeraCom systems. To numerically calculate the PN effect after applying the existing OFDM PN mitigation schemes, a theoretical upper bound for the SINR of the OFDM system affected by PN is adopted, with *K*_0_ indicating the PN floor in dBc/Hz as^52^

19 $${\gamma }_{{\rm{thz}}}=\frac{1}{2(1-{{\rm{e}}}^{-\frac{{K}_{0}B}{4}})+{\gamma }_{0}^{-1}}.$$

#### Consumption factor of TeraCom

As power flows in the typical electronics-based TeraCom system, components IF, PA, BPF, and Mixer (M) consume power, which are represented by their power gain *G*_{PA, BPF, M}_ and efficiency *η*_{PA, BPF, M}_, where efficiency is defined as the ratio of the useful signal power to the total power consumption. Besides, DC power *P*_DC_ is required to drive the LO at both transceivers. Thus, the CF of the TeraCom system is formulated as

20 $${{\rm{CF}}}_{{\rm{thz}}}=\frac{B{\log }_{2}(1+{\gamma }_{{\rm{thz}}})}{\frac{{P}_{{\rm{rec}}}}{{H}_{{\rm{link}}}}+2{P}_{{\rm{DC}}}+{P}_{{\rm{others}}}}.$$

*H*_link_ is then given by the combination of the transceivers’ power efficiency *H*_Tx_ and *H*_Rx_ as^53^

21 $${H}_{{\rm{link}}}^{-1}={H}_{{\rm{Rx}}}^{-1}+\frac{1}{{G}_{{\rm{rec}}}}\left(\frac{1}{{H}_{{\rm{thz}}}}-1\right)+\frac{1}{{G}_{{\rm{rec}}}{H}_{{\rm{thz}}}}\left({H}_{{\rm{Tx}}}^{-1}-1\right),$$

where *G*_rec_ is the receiver gain, and *H*_Tx_ is expressed as

22 $${H}_{{\rm{Tx}}}={\left\{1+\mathop{\sum }\limits_{n = 1}^{N}\frac{1}{\mathop{\prod }\nolimits_{i = n+1}^{N}{G}_{i}}\left(\frac{1}{{\eta }_{n}}-1\right)\right\}}^{-1},$$

where *N* is the number of cascaded components in the transmitter, and in this case, *N* = 4. *i*, *n* ∈ {1, 2, 3, 4} are indices of gain and efficiency vectors $\left\{{G}_{{\rm{PA}}},{G}_{{\rm{BPF1}}},{G}_{{\rm{M}}},{G}_{{\rm{BPF2}}}\right\}$ and $\left\{{\eta }_{{\rm{PA}}},{\eta }_{{\rm{BPF1}}},{\eta }_{{\rm{M}}},{\eta }_{{\rm{BPF2}}}\right\}$. *H*_Rx_ is defined accordingly due to the transceiver asymmetry.

#### Networked systems

As mentioned above, the high directivity elements should be embedded in both TeraCom and VCSEL-based OWC systems to compensate for the severe path loss and obtain better communication performance with energy-concentrated beam transmission. Besides utilizing advanced beam steering techniques, which are still under development for both technologies, multiple access points (APs) are required in a given room to guarantee service coverage. Hence, we derive analytical models for coverage probability and energy efficiency in networked VCSEL-based OWC and TeraCom systems.

The distributed AP deployment scheme is considered here: APs are distributed normally on the room’s ceiling, where the beam direction of each antenna or each VCSEL transmitter is towards the floor, while the receiver’s location is random. For user connection, the nearest AP user association is utilized, which means that the user selects the nearest AP to associate with, and the others are interfering APs.

In networked systems, noise generated by interfering APs should be considered in SINR models. Suppose that there are *N*_t_ × *N*_t_ APs and *N*_r_ receivers, the *l*-th AP and *m*-th receiver form a transmission link ${H}_{{\rm{owc}}}^{(lm)}$ or ${H}_{{\rm{thz}}}^{(lm)}$. For networked-based VCSEL-based OWC systems, Eq. (9) is modified to the SINR of the link between the tagged receiver and its associated AP ${H}_{{\rm{owc}}}^{({l}^{{\prime} }{m}^{{\prime} })}$ as

23 $${\gamma }_{{\rm{owc}}}^{({m}^{{\prime} })}=\frac{{R}_{{\rm{PD}}}^{2}{\left({H}_{{\rm{owc}}}^{({l}^{{\prime} }{m}^{{\prime} })}\right)}^{2}{P}_{{\rm{sig}}}}{\mathop{\sum }\nolimits_{l\ne {l}^{{\prime} }}^{{N}_{{\rm{t}}}\times {N}_{{\rm{t}}}}{R}_{{\rm{PD}}}^{2}{\left({H}_{{\rm{owc}}}^{(l{m}^{{\prime} })}\right)}^{2}{P}_{{\rm{sig}}}+{({\sigma }^{{m}^{{\prime} }})}^{2}},$$

where noise variance is also modified as

24 $$\begin{array}{rcl}{({\sigma }^{{m}^{{\prime} }})}^{2}&=&\frac{4kTB{F}_{{\rm{n}}}}{{R}_{{\rm{L}}}}+2qB\left(\mathop{\sum }\limits_{l=1}^{{N}_{{\rm{t}}}\times {N}_{{\rm{t}}}}{R}_{{\rm{PD}}}{H}_{{\rm{owc}}}^{(l{m}^{{\prime} })}{P}_{{\rm{t}}}\right)\\ &&+B{N}_{{\rm{RI}}}\left(\mathop{\sum }\limits_{l=1}^{{N}_{{\rm{t}}}\times {N}_{{\rm{t}}}}{({R}_{{\rm{PD}}}{H}_{{\rm{owc}}}^{(l{m}^{{\prime} })}{P}_{{\rm{t}}})}^{2}\right).\end{array}$$

Then, the CP of the networked VCSEL-based OWC system is given by

25 $${{\rm{CP}}}_{{\rm{owc}}}({\gamma }_{{\rm{th}}})={\mathbb{P}}({\gamma }_{{\rm{owc}}}^{({m}^{{\prime} })} > {\gamma }_{{\rm{th}}}),$$

where *γ*_th_ indicates threshold SINR and ${\mathbb{P}}(\cdot )$ is the probability density function.

Similarly, interference noises should also be added to the SINR model of TeraCom systems. According to Eq. (19), the SINR of ${m}^{{\prime} }$-th receiver is rewritten as

26 $${\gamma }_{{\rm{thz}}}^{({m}^{{\prime} })}={\left[2\left(1-{{\rm{e}}}^{-\frac{{K}_{0}B}{4}}\right)+\frac{\mathop{\sum }\nolimits_{l\ne {l}^{{\prime} }}^{{N}_{{\rm{t}}}\times {N}_{{\rm{t}}}}{P}_{{\rm{rec}}}^{(l{m}^{{\prime} })}+{\sigma }_{w}^{2}}{{P}_{{\rm{rec}}}^{({l}^{{\prime} }{m}^{{\prime} })}}\right]}^{-1},$$

where ${P}_{{\rm{rec}}}={P}_{{\rm{t}}}{H}_{{\rm{thz}}}^{(l{m}^{{\prime} })}\mathop{\prod }\nolimits_{i = 1}^{N}{G}_{i}$ is related to the transmitted power from the TeraCom transmitter *P*_t_, channel gain, and gains of components at the receiver. CP_thz_(*γ*_th_) is then calculated in the same way as Eq. (25).

To evaluate the energy efficiency of the networked system, the CF of the VCSEL-based OWC is given by

27 $${{\rm{CF}}}_{{\rm{owc}}}=\frac{\mathop{\sum }\nolimits_{m = 1}^{{N}_{{\rm{r}}}}B{\log }_{2}(1+{\gamma }_{{\rm{owc}}}^{(m)})}{\mathop{\sum }\nolimits_{l = 1}^{{N}_{{\rm{t}}}^{2}}\left({P}_{{\rm{T}}}^{(l)}+{P}_{{\rm{others}}}^{(l)}\right)+\mathop{\sum }\nolimits_{m = 1}^{{N}_{{\rm{r}}}}{P}_{{\rm{R}}}^{(m)}}.$$

For the TeraCom networked system, the CF is expressed as

28 $${{\rm{CF}}}_{{\rm{thz}}}=\frac{\mathop{\sum }\nolimits_{m = 1}^{{N}_{{\rm{r}}}}B{\log }_{2}(1+{\gamma }_{{\rm{thz}}}^{(m)})}{\mathop{\sum }\nolimits_{l = 1}^{{N}_{{\rm{t}}}^{2}}\left(\frac{{P}_{{\rm{t}}}^{(l)}}{{H}_{{\rm{Tx}}}}+{P}_{{\rm{DC}}}^{(l)}+{P}_{{\rm{others}}}^{(l)}\right)+\mathop{\sum }\nolimits_{m = 1}^{{N}_{{\rm{r}}}}{P}_{{\rm{DC}}}^{(m)}}.$$

### Outdoor applications: FSO vs. THz

FSO communication and THz are envisioned to play key roles in outdoor applications, i.e., UAV-based, vehicular, and space communications. Channel models for the two technologies have been a research focus in analyzing system performance in outdoor application scenarios. In this section, we adopt widely used stochastic channel models for FSO and TeraCom to compare their performance in various application scenarios. After introducing the channel models, we specify the use cases of UAVs.

#### FSO stochastic channel model

FSO channel gain consists of four impairments: i.e., atmospheric path loss *h*_l_, turbulence-induced fading *h*_t_, pointing errors *h*_p_, and angle of arrival (AoA) fluctuations induced link interruption *h*_a_; thus, the channel gain is given by

29 $${h}_{{\rm{fso}}}={h}_{{\rm{l}}}{h}_{{\rm{t}}}{h}_{{\rm{p}}}{h}_{{\rm{a}}}.$$

The atmospheric path loss for the FSO link is expressed below according to Beer–Lambert law

30 $${h}_{{\rm{l}}}=\exp (-{\xi }_{{\rm{l}}}L),$$

where *ξ*_l_ is the attenuation factor closely related to the weather condition and *L* is the link length. The effects of turbulence and misalignment are modeled by statistical processes, which are detailed in the following.

Málaga distribution is a general form, as other commonly used distributions, e.g., the Gamma–Gamma distribution, are its special cases. The probability density function (PDF) of the Málaga distribution, which is used to model turbulence-induced fading, is given by

31 $${f}_{{h}_{{\rm{t}}}}({h}_{{\rm{t}}})={A}_{M}\mathop{\sum }\limits_{s=1}^{\beta }{a}_{s}{h}_{{\rm{t}}}^{\frac{\epsilon +\beta }{2}-1}{K}_{\epsilon -\beta }\left(s\sqrt{\frac{\epsilon \beta {h}_{{\rm{t}}}}{g\beta +{\Omega }^{{\prime} }}}\right),$$

where the key factors are modeled as

32 $$\left\{\begin{array}{l}{A}_{M}=\frac{2{\epsilon }^{\epsilon /2}}{{g}^{1+\epsilon /2}\Gamma (\epsilon )}{\left(\frac{g\beta }{g\beta +{\Omega }^{{\prime} }}\right)}^{\beta +\epsilon /2}\quad \\ {a}_{s}=\left(\begin{array}{c}\beta -1\\ s-1\end{array}\right)\frac{{\left(g\beta +{\Omega }^{{\prime} }\right)}^{1-s/2}}{(s-1)!}{\left(\frac{{\Omega }^{{\prime} }}{g}\right)}^{s-1}{\left(\frac{\epsilon }{\beta }\right)}^{s/2}\quad \\ g=2{b}_{0}(1-\rho ),{\Omega }^{{\prime} }=\Omega +2{b}_{0}\rho +2\sqrt{2{b}_{0}\rho \Omega }\cos \left({\varphi }_{a}-{\varphi }_{b}\right)\quad \end{array}\right.,$$

where *K*_*ϵ*−*β*_ indicates the modified Bessel function of the second kind of (*ϵ* − *β*)th-order, and *Γ*( ⋅ ) indicates the Gamma function. *Ω*, *φ*_*a*_, *φ*_*b*_ represent the LoS component’s average power, deterministic phase, and the deterministic phase of the coupled-to-LoS scatter component, respectively; 2*b*_0_ and 0 < *ρ* < 1 denote the average power of the total scatter components and the amount of scattering power coupled to the LoS component. *ϵ* and *β* are large-scale and small-scale scattering parameters that rely on Rytov variance, link distance, and refractive index structure parameter^54^.

The misalignment effect for the FSO link includes pointing errors and AoA fluctuation-induced link interruption. Considering the Gaussian beam profile at the receiving end, the PDF of pointing errors, *h*_p_ is

33 $${f}_{{h}_{{\rm{p}}}}({h}_{{\rm{p}}})=\frac{{\xi }_{{\rm{p}}}^{2}}{{A}_{0}^{{\xi }_{{\rm{p}}}^{2}}}{h}_{{\rm{p}}}^{{\xi }_{{\rm{p}}}^{2}-1},\quad 0\le {h}_{{\rm{p}}}\le {A}_{0},$$

where *ξ*_p_ = *w*_e_/(2*σ*_m_) with *w*_e_ denoting the equivalent beam waist at the receiver side and *σ*_m_ is the total displacement square variance. *A*_0_ is the fraction of power collected at the detector without pointing errors. The above parameters are obtained as follows

34 $${w}_{{\rm{e}}}=\frac{{w}_{{\rm{z}}}^{2}\sqrt{2}\,\text{erf}\,(v)}{2v\exp \left(-{v}^{2}\right)},\quad {A}_{0}=\,\text{erf}\,{(v)}^{2},\quad v=\sqrt{\frac{\pi }{2}}\frac{{r}_{{\rm{a}}}}{{w}_{{\rm{z}}}},$$

where *r*_*a*_ is the aperture radius of the receiver, and *w*_z_ is the beam waist of the Gaussian beam at the receiver, which is given by^47^

35 $${w}_{{\rm{z}}}={w}_{0}\sqrt{1+\left(1+\frac{2{w}_{0}^{2}}{{(0.55{C}_{n}^{2}{k}_{f}^{2}L)}^{-3/5}}\right){\left(\frac{L}{{z}_{{\rm{R}}}}\right)}^{2}},$$

where ${C}_{n}^{2}$ is the refractive index structure parameter, and *k*_*f*_ = 2*π*/*λ* is the beam wave number.

Meanwhile, the PDF of AoA fluctuation is expressed as

36 $${f}_{{h}_{{\rm{a}}}}({h}_{{\rm{a}}})={a}_{1}\delta ({h}_{{\rm{a}}})+(1-{a}_{1})\delta ({h}_{{\rm{a}}}-1),\quad {a}_{1}=\exp \left(\frac{-{\theta }_{{\rm{FoV}}}^{2}}{{a}_{2}{\sigma }_{a}^{2}}\right),$$

where *δ*( ⋅ ) is the Dirac delta function, *θ*_FoV_ is the FoV of the receiver, *σ*_*a*_ is the AoA fluctuation square variance, and *a*_2_ = 2 or *a*_2_ = 4 satisfy the situation where one or two sides have vibrations.

Considering intensity modulation direct detection (IM/DD) for the FSO system and the above key parts in the FSO channel model, the outage probability is

37 $${F}_{{\gamma }_{{\rm{fso}}}}({\gamma }_{{\rm{th}}})\approx {a}_{1}+\left(1-{a}_{1}\right)D\times \mathop{\sum }\limits_{s=1}^{\beta }{c}_{s}G\begin{array}{l}6,1\\ 3,7\end{array}\left({A}_{1}\left\vert \,\begin{array}{l}1,{K}_{1}\\ {K}_{2},0\end{array}\right.\right),$$

where the details can be found as^47^

38 $$\left\{\begin{array}{ll}D={\xi }_{{\rm{p}}}^{2}{A}_{M}/(8\pi ),\quad {c}_{s}={2}^{\epsilon +s-1}{b}_{s},\quad {b}_{s}={a}_{s}{\left(\epsilon \beta /\left(g\beta +{\Omega }^{{\prime} }\right)\right)}^{-(\epsilon +s)/2},\quad \\ {A}_{1}=\frac{{B}^{2}{\gamma }_{{\rm{th}}}}{16{\overline{\gamma }}_{{\rm{fso}}}},\,B=\frac{{\xi }_{{\rm{p}}}^{2}(g+{\Omega }^{{\prime} })(1-{a}_{1})\epsilon \beta }{({\xi }_{{\rm{p}}}^{2}+1)(g\beta +{\Omega }^{{\prime} })},\quad \\ {K}_{1}=\left[({\xi }_{{\rm{p}}}^{2}+1)/2,({\xi }_{{\rm{p}}}^{2}+2)/2\right],\quad {K}_{2}=\left[\frac{{\xi }_{{\rm{p}}}^{2}}{2},\frac{{\xi }_{{\rm{p}}}^{2}+1}{2},\frac{\epsilon }{2},\frac{\epsilon +1}{2},\frac{s}{2},\frac{s+1}{2}\right],\quad \end{array}\right.$$

where ${\overline{\gamma }}_{{\rm{fso}}}$ and *γ*_th_ denote the average and threshold SNR of the FSO link, and ${G}_{p,q}^{m,n}(\cdot )$ is the Meijer G-function.

#### THz stochastic channel model

THz channel gain includes the effect of path loss *g*_l_, multi-path fading *g*_t_, and misalignment *g*_p_, which is given by

39 $${h}_{{\rm{thz}}}={g}_{{\rm{l}}}{g}_{{\rm{t}}}{g}_{{\rm{p}}}.$$

As introduced in Eq. (14), the pass loss in the THz link is described as ${g}_{{\rm{l}}}=\sqrt{{G}_{0}^{{\rm{Tx}}}{G}_{0}^{{\rm{Rx}}}}\frac{c}{4\pi fL}\exp [\frac{1}{2}\kappa (f)L]$, with *L* indicating the link length. *κ*(*f*) depends on RH. ${G}_{0}^{{\rm{Tx}}}$ and ${G}_{0}^{{\rm{Rx}}}$ denote the maximum antenna gains at the transmitter and receiver if there is no pointing error. As noted in the “Comparative overview of THz and optical wireless systems” section, while atmospheric turbulence considerably impacts FSO links due to their short wavelengths, its effect on THz links is negligible owing to the reduced sensitivity of longer wavelengths to refractive index fluctuations.

*α* − *μ* distribution is widely adopted to model the multi-path fading for THz links, where the PDF of *g*_t_ reads^55^

40 $${f}_{{g}_{{\rm{t}}}}\left({g}_{{\rm{t}}}\right)=\frac{\alpha {\mu }^{\mu }}{{\hat{{g}_{{\rm{t}}}}}^{\alpha \mu }\Gamma (\mu )}{g}_{{\rm{t}}}^{\alpha \mu -1}\exp \left(-\mu \frac{{g}_{{\rm{t}}}^{\alpha }}{{\hat{{g}_{{\rm{t}}}}}^{\alpha }}\right),$$

where *α*, *μ* denote the fading parameter and normalized variance of the fading channel envelope, respectively, and $\hat{{g}_{{\rm{t}}}}$ is the *α*-root mean value of the fading channel envelope.

For THz links, pointing errors are modeled with different methods. One widely used method is adopting the same pointing error model as in the FSO link, where the misalignment effect is demonstrated by the power loss due to the displacement of the THz Gaussian beam spot and the THz receiver center. Then, the outage probability of the THz link is given by^56^

41 $${F}_{{\gamma }_{{\rm{thz}}}}({\gamma }_{{\rm{th}}})=\frac{{C}_{1}{C}_{2}}{\alpha }\left[{\gamma }_{{\rm{th}}}^{\frac{{\xi }_{{\rm{p}}}^{2}}{2}}/{\bar{\gamma }}_{{\rm{thz}}}^{\frac{{\xi }_{{\rm{p}}}^{2}}{2}}\right]\times {G}_{2,3}^{2,1}\left({C}_{{T}_{1}}{\gamma }_{{\rm{th}}}^{\frac{\alpha }{2}}/{\bar{\gamma }}_{{\rm{thz}}}^{\frac{\alpha }{2}}\left|\,\begin{array}{c}1-{\xi }_{{\rm{p}}}^{2}/\alpha ,1\\ 0,{C}_{{T}_{2}},-{\xi }_{{\rm{p}}}^{2}/\alpha \end{array}\right.\right)$$

Notations used in the expression are outlined by

42 $${C}_{1}=\frac{{\xi }_{{\rm{p}}}^{2}}{{A}_{0}^{{\xi }_{{\rm{p}}}^{2}}},{C}_{2}=\frac{{\mu }^{{\xi }_{{\rm{p}}}^{2}/\alpha }}{{\hat{{g}_{{\rm{t}}}}}^{{\xi }_{{\rm{p}}}^{2}}\Gamma (\mu )},{C}_{{T}_{1}}=\frac{\mu }{{A}_{0}^{\alpha }{\hat{{g}_{{\rm{t}}}}}^{\alpha }},{C}_{{T}_{2}}=\frac{\alpha \mu -{\xi }_{{\rm{p}}}^{2}}{\alpha },$$

where *ξ*_p_ and *A*_0_ are modeled similarly to the FSO link, i.e., *ξ*_p_ is the ratio of equivalent beam waist to the displacement square variance, and *A*_0_ is represented in Eq. (34).

Recently, research showed that the misalignment effect model in THz links should be different from FSO links, where the antenna pattern is the key impact factor^23^. The antenna gain is included in the pointing error model, not the path loss model; thus, with this derivation ${g}_{{\rm{l}}}^{{\prime} }=\frac{c}{4\pi fL}\exp [\frac{1}{2}\kappa (f)L]$. The normalized pointing error is stated as

43 $${g}_{{\rm{p}}}=\sqrt{{G}^{{\rm{Tx}}}({\theta }_{{\rm{ta}}},{\theta }_{{\rm{te}}})}\sqrt{{G}^{{\rm{Rx}}}({\theta }_{{\rm{ra}}},{\theta }_{{\rm{re}}})},$$

where *G*^Tx/Rx^ denotes the transceivers’ normalized antenna radiation pattern with azimuth/elevation angle of antenna pointing direction at the transmitter side *θ*_ta_, *θ*_te_, and antenna pointing angles at the receiver side *θ*_ra_, ${\theta }_{{\rm{re}}}$. For simplicity, the above variations are characterized as ${\theta }_{{\rm{ta}}} \sim {\mathcal{N}}(0,{\sigma }_{\theta })$, ${\theta }_{{\rm{te}}} \sim {\mathcal{N}}(0,{\sigma }_{\theta })$, ${\theta }_{{\rm{ra}}} \sim {\mathcal{N}}(0,{\sigma }_{\theta })$, and ${\theta }_{{\rm{re}}} \sim {\mathcal{N}}(0,{\sigma }_{\theta })$. Then, the PDF of the pointing error is formulated as^48^

44 $${f}_{{g}_{{\rm{p}}}}\left({g}_{{\rm{p}}}\right)=\frac{{\xi }^{2}}{{G}_{0}^{\xi }}\ln \left({G}_{0}\right)\times {g}_{{\rm{p}}}^{\xi -1}-\frac{{\xi }^{2}}{{G}_{0}^{\xi }}\ln \left({g}_{{\rm{p}}}\right)\times {g}_{{\rm{p}}}^{\beta -1},$$

where $\xi =\frac{{\omega }_{B}^{2}}{{\sigma }_{\theta }^{2}}$. *G*_0_ and *ω*_*B*_ are antenna number-related antenna gain and HPBW, i.e., ${G}_{0}({N}_{{\rm{ant}}})=\pi {N}_{{\rm{ant}}}^{2}$ and *ω*_*B*_(*N*_ant_) = 1.061/*N*_ant_ with a uniform linear array (ULA) antenna. Then, considering both the multi-path fading and misalignment effect, the CDF of the THz link *h*_thz_ is modeled as

45 $$\begin{array}{ll}{F}_{{h}_{{\rm{thz}}}}({h}_{{\rm{thz}}})\,=\,1-\mathop{\sum }\limits_{k=0}^{\mu -1}\frac{{B}_{1}{h}_{{\rm{thz}}}}{\alpha }\frac{a{\xi }^{2}}{\Gamma (k+1)}\left[{\left({B}_{1}{h}_{{\rm{thz}}}\right)}^{{B}_{2}}{e}^{-\left(\frac{{\left.{B}_{1}{h}_{{\rm{thz}}}\right)}^{\alpha }}{2}\right)}\right.\\\qquad\qquad\,\,\left.{{\mathbb{W}}}_{{B}_{3},{B}_{4}}\left({B}_{1}^{\alpha }{h}_{{\rm{thz}}}^{\alpha }\right)-{\left({B}_{1}{h}_{{\rm{thz}}}\right)}^{{B}_{5}}{e}^{-\left(\frac{{\left({B}_{1}{h}_{{\rm{thz}}}\right)}^{\alpha }}{2}\right)}{{\mathbb{W}}}_{{B}_{6},{B}_{7}}\left({B}_{1}^{\alpha }{h}_{{\rm{thz}}}^{\alpha }\right)\right]\end{array}$$

with the key notations given by

46 $$\left\{\begin{array}{ll}{B}_{1}=\frac{{\mu }^{1/\alpha }}{{G}_{0}{\hat{g}}_{t}{g}_{{\rm{l}}}^{{\prime} }},{B}_{2}=\frac{\alpha (k-1)}{2}+\frac{\xi }{2}-\frac{1}{2a}-1,\quad \\ {B}_{3}=\frac{k-1}{2}-\frac{\xi }{2\alpha }+\frac{1}{2a\alpha },{B}_{4}={B}_{3}+\frac{1}{2},\quad \\ {B}_{5}=\frac{\alpha (k-1)}{2}+\frac{\xi }{2}-1,{B}_{6}=\frac{k-1}{2}-\frac{\xi }{2\alpha },{B}_{7}={B}_{6}+\frac{1}{2}\quad \end{array}\right.$$

where *a* is a large number to approximate $\ln ({g}_{{\rm{p}}})$, ${{\mathbb{W}}}_{\cdot ,\cdot }\left(\cdot \right)$ indicates Whittaker, and function *Γ*( ⋅ ) represents the incomplete gamma function. Denoting ${h}_{{\rm{thz}}}={g}_{{\rm{l}}}^{{\prime} }\sqrt{\frac{{\gamma }_{{\rm{th}}}}{{\overline{\gamma }}_{{\rm{thz}}}}}$, we can obtain the outage probability of the THz link as ${F}_{{h}_{{\rm{thz}}}}\left({g}_{{\rm{l}}}^{{\prime} }\sqrt{\frac{{\gamma }_{{\rm{th}}}}{{\overline{\gamma }}_{{\rm{thz}}}}}\right)$.

#### Comparison of UAV applications

In outdoor environments, UAV-based THz/FSO links introduce unique challenges compared to terrestrial systems due to their dynamic nature and susceptibility to aerial platform instabilities. We analyze three key link scenarios^25,26,47^:

- U2U: Both the transmitter and receiver experience mobility-induced 6-degrees of freedom (DoF) fluctuations.
- U2G: Downlink where the hovering instability of the UAV dominates.
- G2U: Uplink with ground station stability, but UAV receiver motion.

To analyze the FSO performance with the three above links, considering the misalignment due to the movement of UAVs, the key is to model the total displacement square variance *σ*_m_ and the AoA fluctuation square variance *σ*_a_. With Cartesian coordinates, we denote *σ*_*txp*_, *σ*_*typ*_ as the standard deviation (SD) of transmitter position in the *x* − *z* and *y* − *z* plane, *σ*_*rxp*_, *σ*_*ryp*_ as the SD of the receiver position, *σ*_*txo*_, *σ*_*tyo*_ as the SD of the transmitter orientation, and *σ*_*rxo*_, *σ*_*ryo*_ as the SD of the receiver orientation, respectively. Besides, a non-zero foresight angle of the transmitter and receiver UAVs is considered, which is denoted by *μ*_*tx*_, *μ*_*ty*_, *μ*_*rx*_, *μ*_*ry*_, respectively^25,47^. Then, the total displacement variance for the three links is

47 $${\sigma }_{{\rm{m}}}^{2}=\left\{\begin{array}{ll}{\left(\frac{3{Z}^{2}{\mu }_{tx}^{2}{\sigma }_{dx}^{4}+3{Z}^{2}{\mu }_{ty}^{2}{\sigma }_{dy}^{4}+{\sigma }_{dx}^{6}+{\sigma }_{dy}^{6}}{2}\right)}^{\frac{1}{3}},\quad &\,\text{U2U}\,\,\& \,\,\text{U2G}\,\\ {\sigma }_{txp}^{2}+{\sigma }_{rxp}^{2}+{\sigma }_{typ}^{2}+{\sigma }_{ryp}^{2},\quad &\,\text{G2U}\,\end{array}\right.$$

where ${\sigma }_{dx}^{2}={L}^{2}{\sigma }_{txo}^{2}+{\sigma }_{txp}^{2}+{\sigma }_{rxp}^{2}$ and ${\sigma }_{dy}^{2}={L}^{2}{\sigma }_{tyo}^{2}+{\sigma }_{typ}^{2}+{\sigma }_{ryp}^{2}$. AoA fluctuation variance is

48 $${\sigma }_{a}^{2}=\left\{\begin{array}{l}{\left(\frac{3{\mu }_{x}^{2}{\sigma }_{x}^{4}+3{\mu }_{y}^{2}{\sigma }_{y}^{4}+{\sigma }_{x}^{6}+{\sigma }_{y}^{6}}{2}\right)}^{\frac{1}{3}},\,\text{U2U}\,\quad \\ {\left(\frac{3{\mu }_{tx}^{2}{\sigma }_{txo}^{4}+3{\mu }_{ty}^{2}{\sigma }_{tyo}^{4}+{\sigma }_{txo}^{6}+{\sigma }_{tyo}^{6}}{2}\right)}^{\frac{1}{3}},\,\text{U2G}\,\quad \\ {\left(\frac{3{\mu }_{rx}^{2}{\sigma }_{rxo}^{4}+3{\mu }_{ry}^{2}{\sigma }_{ryo}^{4}+{\sigma }_{rxo}^{6}+{\sigma }_{ryo}^{6}}{2}\right)}^{\frac{1}{3}},\,\text{G2U}\,\quad \end{array}\right.$$

where *μ*_*x*_ = *μ*_*tx*_ + *μ*_*rx*_, *μ*_*y*_ = *μ*_*ty*_ + *μ*_*ry*_, ${\sigma }_{x}^{2}={\sigma }_{txo}^{2}+{\sigma }_{rxo}^{2}$, and ${\sigma }_{y}^{2}={\sigma }_{tyo}^{2}+{\sigma }_{ryo}^{2}$. From Eqs. (47 and 48), the combined displacement variance ${\sigma }_{{\rm{m}}}^{2}$ and AoA variance ${\sigma }_{{\rm{a}}}^{2}$ both follow the generalized cube-mean expressions, yielding heavier tails in the pointing error distribution than in any terrestrial case. Both U2G and G2U scenarios exhibit lower AoA fluctuation variance than the U2U link. Moreover, owing to the ground station’s inherent stability and its reduced susceptibility to motion-induced misalignment, the G2U configuration yields the smallest displacement variance. With the model in Eq. (45) for UAV-based THz links, we use *σ*_m_ divided by link length to denote the total pointing angle error square variance.

## Data Availability

All relevant data and figures supporting the main conclusions of the document are available on request. Please refer to Mingqing Liu at clare@tongji.edu.cn.

## Code availability

All relevant code supporting the document is available upon request. Please refer to M.L. at clare@tongji.edu.cn.
